# Developmental normalization of phenomics data generated by high throughput plant phenotyping systems

**DOI:** 10.1186/s13007-020-00653-x

**Published:** 2020-08-12

**Authors:** Diego Lozano-Claros, Xiangxiang Meng, Eddie Custovic, Guang Deng, Oliver Berkowitz, James Whelan, Mathew G. Lewsey

**Affiliations:** 1 Department of Animal, Plant and Soil Science, AgriBio Building, La Trobe University, Bundoora, VIC 3086 Australia; 2 Department of Engineering, School of Engineering and Mathematical Sciences, La Trobe University, Melbourne, VIC 3086 Australia; 3 Australian Research Council Research Hub for Medicinal Agriculture, AgriBio Building, La Trobe University, Bundoora, VIC 3086 Australia; 4 Australian Research Council Centre of Excellence in Plant Energy Biology, La Trobe University, Bundoora, VIC 3086 Australia; 5 Currently: Key Laboratory of Biofuels, Shandong Provincial Key Laboratory of Energy Genetics, Qingdao Institute of Bioenergy and Bioprocess Technology, Chinese Academy of Sciences, Qingdao, 266101 China

**Keywords:** High-throughput plant phenotyping, Development, Growth, Computer vision, Machine learning, Phenomics

## Abstract

**Background:**

Sowing time is commonly used as the temporal reference for *Arabidopsis thaliana* (Arabidopsis) experiments in high throughput plant phenotyping (HTPP) systems. This relies on the assumption that germination and seedling establishment are uniform across the population. However, individual seeds have different development trajectories even under uniform environmental conditions. This leads to increased variance in quantitative phenotyping approaches. We developed the Digital Adjustment of Plant Development (DAPD) normalization method. It normalizes time-series HTPP measurements by reference to an early developmental stage and in an automated manner. The timeline of each measurement series is shifted to a reference time. The normalization is determined by cross-correlation at multiple time points of the time-series measurements, which may include rosette area, leaf size, and number.

**Results:**

The DAPD method improved the accuracy of phenotyping measurements by decreasing the statistical dispersion of quantitative traits across a time-series. We applied DAPD to evaluate the relative growth rate in Arabidopsis plants and demonstrated that it improves uniformity in measurements, permitting a more informative comparison between individuals. Application of DAPD decreased variance of phenotyping measurements by up to 2.5 times compared to sowing-time normalization. The DAPD method also identified more outliers than any other central tendency technique applied to the non-normalized dataset.

**Conclusions:**

DAPD is an effective method to control for temporal differences in development within plant phenotyping datasets. In principle, it can be applied to HTPP data from any species/trait combination for which a relevant developmental scale can be defined.

## Background

Sowing-time is often taken as the initial time point for measuring plant phenotypic traits in HTPP systems [1]. Traits of a number of plants are typically measured between 2 defined timepoints, chosen relative to the time of sowing. However, the germination of individual seeds in a genetically identical population is normally distributed, even under uniform conditions [2]. Differences in the timing of germination and seed establishment increase the dispersion of time-series measurements because individual plants will be at different developmental stages at any given time point. This may create difficulty in drawing reliable conclusions from the data.

Data normalization is often applied to analyze datasets that have high dispersion, but many methods are not suitable for time-series plant phenotyping data. Traditional methods such as the z-score, min–max, and decimal scaling are not appropriate because of statistical parameters such as mean and standard deviation change over time. Time-series normalization methods can numerically fit the time-series measurements to a single timeline and reduce dispersion. However, these methods do not take into consideration developmental information nor the effect of the allometric scaling of growth; individual seedlings can have similar trait values but may be at different developmental stages.

Plants progress through specific developmental stages that are consistent between genetically identical individuals grown in uniform conditions. For example, the adjusted BASF, Bayer, Ciba-Geigy (BBCH) scale describes Arabidopsis developmental stages using seed germination, leaf development, rosette growth, inflorescence emergence, flower production, silique ripening and senescence as significant markers [3]. Considering the availability of clear developmental stage scales, development normalization could be an appropriate method to normalize HTPP data. Developmental normalization would arrange time-series plant phenotype measurements based upon plants being at similar developmental stages. However, developmental normalization has not previously been implemented in an automated manner suitable for image-based HTPP, likely due to the technical difficulty. To do so requires the integration of methods that quantitatively measure growth and developmental traits in time-series for hundreds of plants, detect defined developmental stages, and shift the time-series data for each plant independently such that developmental stages align across all plants without distortion of the underlying growth trends.

Image-based plant phenotyping systems are now widely used [4]. However, image processing remains one of the most considerable difficulties for these systems, especially image segmentation of shoots and leaves. The performance depends heavily on the complexity of images, which frequently include interference, light reflection, leaf overlap, and foreign objects that must be removed (for example, pots, and soil) [5]. Furthermore, the identification of multiple leaves at the same time (multi-instance segmentation) is difficult due to their similarity in shape and appearance [6]. Without algorithms that extract accurate measurements, it is more complicated to scale the principal environmental variables influencing the phenotype and underlying physiological processes [7].

Several approaches have been developed to address the challenge of image segmentation. They can be categorized into 4 groups; shape analysis, watershed-based, machine learning, and graph-based. Shape analysis algorithms rely on assumptions regarding plant geometrical features and structure, but they may fail when encountering new data, which limits their applicability [8, 9]. Watershed-based methods consider a grey image as a topographic surface produced by its intensity gradients, where light pixels are represented as high-intensity values and dark pixels as low-intensity values [4]. However, the performance of watershed algorithms is compromised due to over-segmentation when leaves overlap. Machine learning segmentation approaches can be unsupervised or supervised. Unsupervised learning algorithms are mainly used for pixel clustering. They identify individual leaves by grouping pixels which share a similar feature pattern such as color, texture, and others. Supervised learning algorithms analyze and compare the input plant images with annotated images or labels [10]. Graph-based methods segment individual leaves by applying graph-based noise removal and region growing techniques [8].

We developed DAPD, which combines time-series trait measurement with normalization by developmental stage in an automated manner. DAPD synchronizes the start-point of timelines for all plants in the population by their number of leaves. To achieve this, we also developed a new leaf segmentation algorithm that utilizes a decision tree algorithm that selects the most suitable algorithm from multiple image processing algorithms based on image features. These algorithms include K-means, image histogram analysis, and shape analysis [11–13]. Also, the marker-controlled watershed algorithm was used for individual leaf segmentation [14]. This combined approach overcomes the limitations of using a single segmentation method. We applied DAPD to evaluate the rosette area in Arabidopsis and demonstrated that it improved uniformity in measurements, enabling a more informative comparison between individuals. The algorithm allows users to select and define the starting leaf number relevant to their experiment. Our code and a Python notebook come with a friendly user manual detailing how to use it, and they are available at https://github.com/diloc/DAPD_Normalization.git.

## Results

### DAPD time normalization of plant phenomics data

Our major aim was to produce an automated method for the developmental stage synchronization of HTPP data. To achieve this, we developed DAPD, which synchronizes shoot phenotypic measurements of multiple Arabidopsis plants by normalizing time-series measurements to a reference time point (i.e., a day). DAPD does so by identifying the highest cross-correlation score between leaf number and day-after-sowing (DAS) of seedlings of the same genotype. First, seedlings were grouped by genotype, then their leaf number was assessed by applying the DAPD-Leaf Counting algorithm. Next, a daily leaf number was associated with a specific day in the DAS timeline, and the degree of similarity between them was calculated by cross-correlation. The highest score indicates the day where the leaf number of time-series are best aligned. We intended that the application of DAPD would decrease the dispersion of time-series phenotype measurements.

**Fig. 1:**
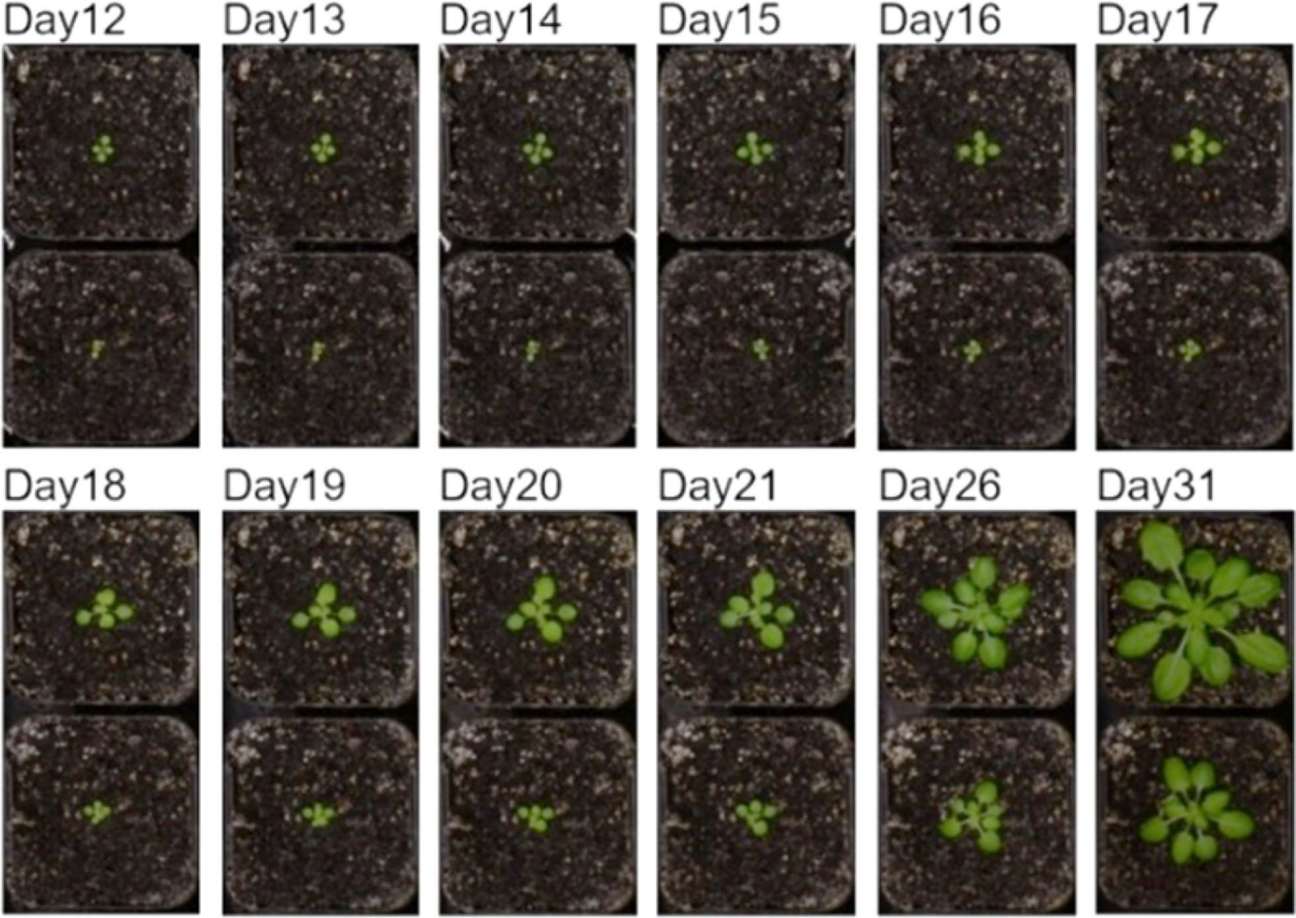
Developmental differences in 2 neighboring Arabidopsis seedlings (ecotype Col-0) grown under uniform environmental conditions

We tested DAPD on 2 independent experiments comprised of many Arabidopsis ecotype Col-0 and Cvi plants. In the first experiment, we used 355 individuals of Col-0 only and, in the second experiment, 140 individuals of each ecotype. These plants were imaged every 10–15 min during the daytime from 12 to 32 days after sowing. Replicate plants of the same ecotype within experiments were at different developmental stages, as assessed by leaf number, despite being sown at the same time and having been grown from seeds of plants cultivated under conditions that would not result in seed dormancy (Fig. 1).

**Fig. 2:**
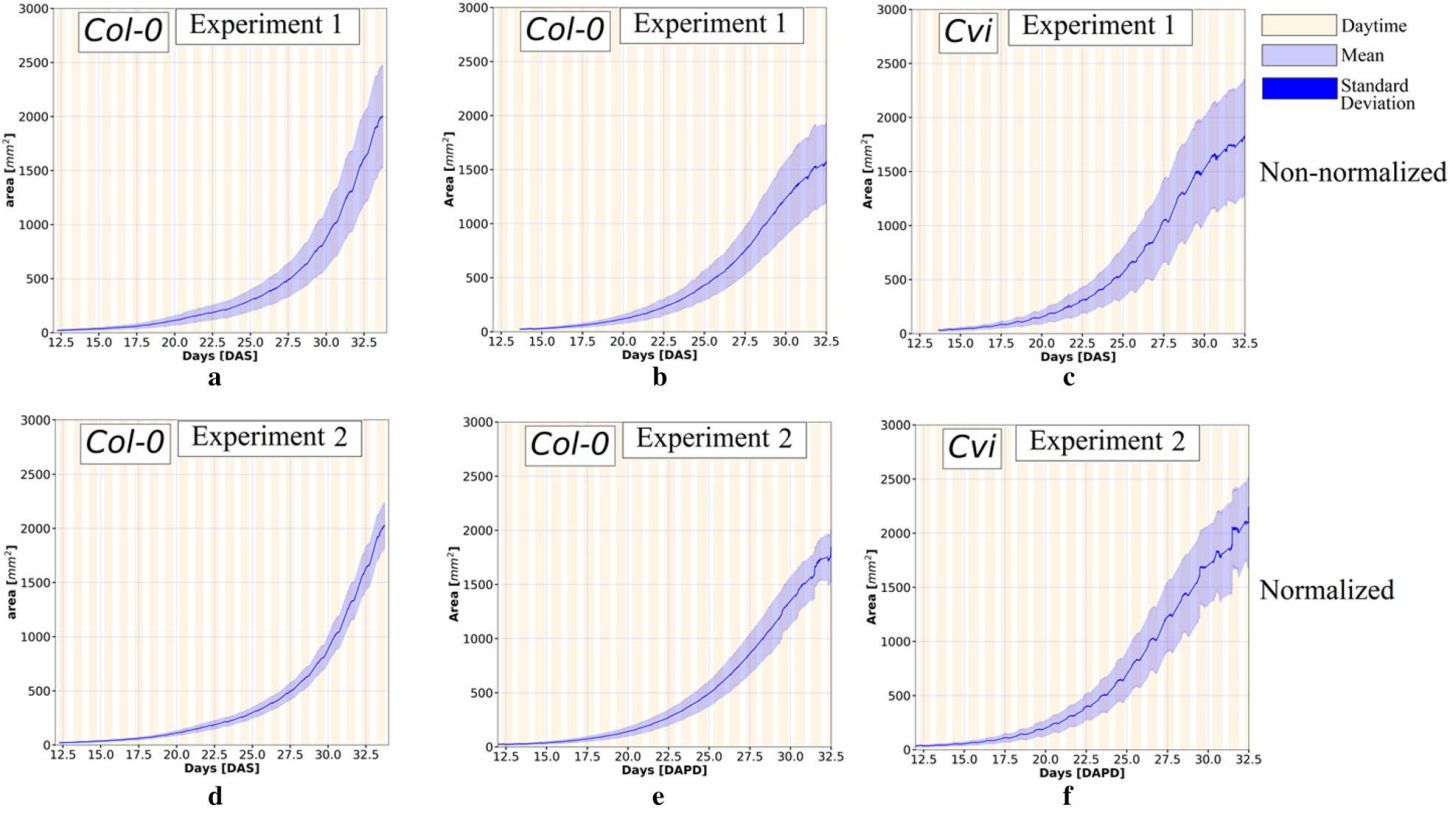
Mean and standard deviation of the non-normalized and normalized projected rosette area datasets. The top row shows the non-normalized datasets: (**a**) Col-0 plants in experiment 1, (**b**) Col-0 plants in experiment 2, and (**c**) Cvi plants in experiment 2. The bottom row shows the normalized datasets: (**d**) Col-0 plants in experiment 1, (**e**) Col-0 plants in experiment 2, and (**f**) Cvi plants in experiment 2. The light purple strip indicates the standard deviation and the solid blue curve in the mean area

The rosette area measurements of all individual Col-0 and Cvi plants were extracted from the images. The mean and standard deviation of these non-normalized measurements were calculated (Fig. 2). The overall dispersion of the non-normalized datasets consistently grew from day 12 to day 32, as assessed from the standard deviation of the rosette area measurements (Fig. 2a–c).

DAPD was applied to normalize the Col-0 and Cvi rosette area datasets. The standard deviation and mean values were calculated at multiple time points to assess the dispersion of the data (Fig. 2d–f). The normalized data preserved the exponential growth pattern observed in the non-normalized data, but the overall statistical dispersion of the normalized data was considerably smaller. Daily oscillations in leaf area were also observed, most notably in the Cvi dataset. They occurred due to the diurnal change in the elevation angle of Arabidopsis leaves, which increases and decreases the perspective of the leaves relative to the cameras. Notably, DAPD preserves these signals post-normalization because it shifts the time-series in whole day increments.

**Fig. 3:**
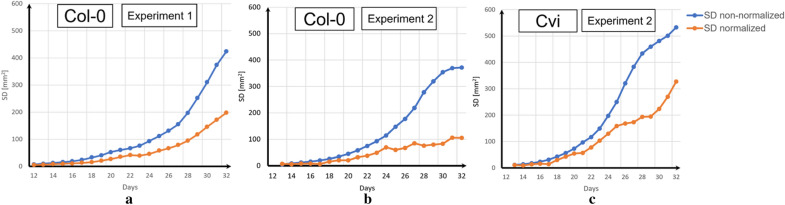
Standard deviation (SD) comparison between the non-normalized and normalized datasets. **a** Col-0 plants in experiment 1, **b** Col-0 plants in experiment 2, and **c** Cvi plants experiment 2

Examining the standard deviation over the time-series confirmed the observation that DAPD time normalization reduces the dispersion of rosette area measurements (Fig. 3). During the period from day 13 to day 32, the standard deviation of the non-normalized dataset exponentially increased in the three datasets whilst the standard deviation of the normalized dataset linearly increased. DAPD normalization reduced the dispersion of the measurements at day 32 by between 1.5 and 3.5 times.

### DAPD detects outliers with different growth traits amongst a population

**Fig. 4:**
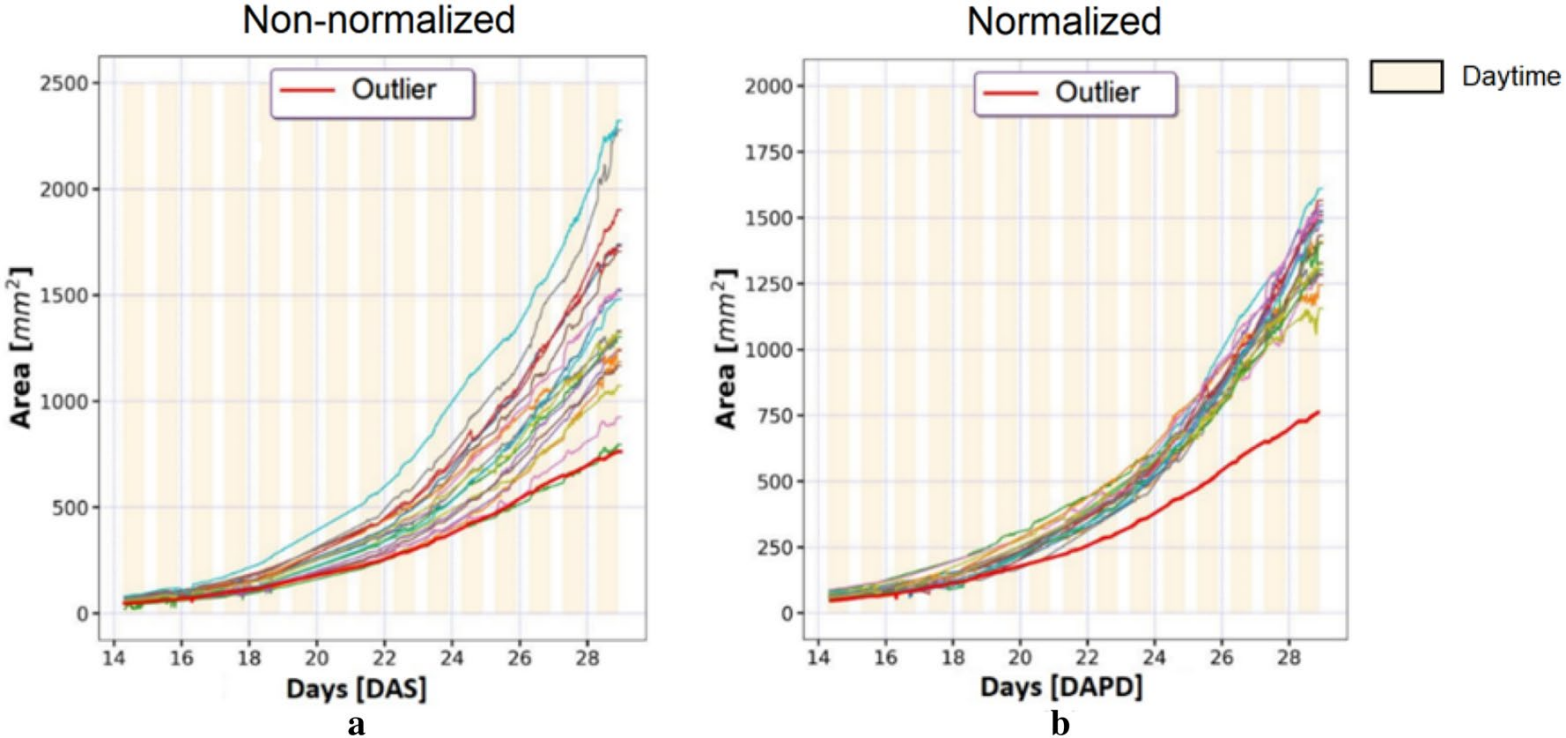
Non-normalized and normalized rosette area curves of 24 Col-0 individuals in experiment 1, (**a**) non-normalized Col-0 curves, and (**b**) normalized Col-0 curves. The red and thick curve represents the rosette area of a pathogen-infected individual (outlier) before and after normalization

**Fig. 5:**
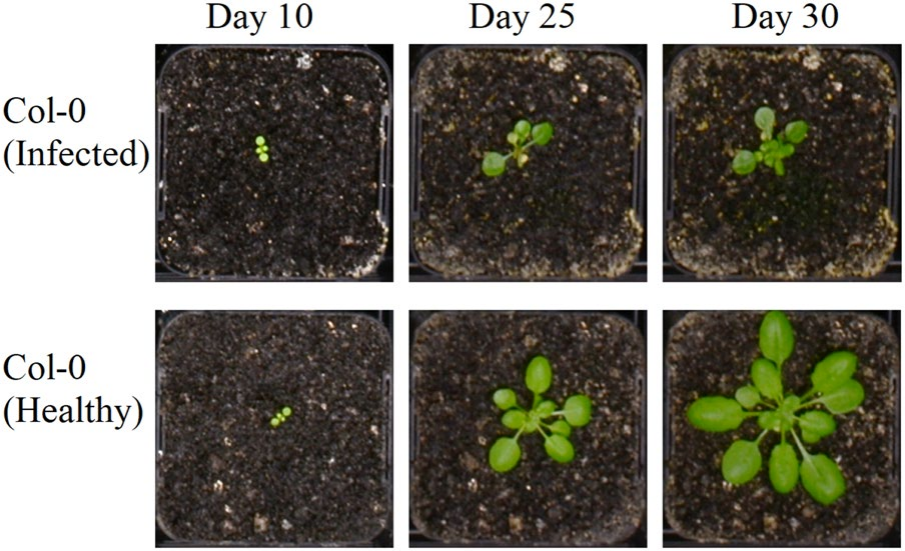
A pathogen-infected plant with outlier growth traits detected automatically using DAPD. The pathogen-infected plant had a smaller number of leaves and rosette area than the healthy plant

A powerful application of DAPD is the detection of outliers in HTPP datasets. It is common in large-scale phenomics experiments to observe a small number of individual plants that develop abnormally, despite being of the same genotype as all other members of the population and being grown in uniform conditions. These individuals may have been affected by unintended stresses, and, in some situations, it is reasonable to remove them from datasets. We assessed the ability of DAPD to detect outliers on 24 wild-type Col-0 individuals grown 2 different trays (Fig. 4). It was difficult to confidently identify outliers amongst the non-normalized growth curves by visual inspection or application of a central tendency metric (mean, median, mode of rosette area). However, after applying DAPD normalization, a putative outlier was identified clearly. This plant did not follow the same growth trajectory as the rest of the population, having a smaller rosette area from day 20 onwards. A visual inspection determined that this plant was infected by a pathogen (Fig. 5). These results demonstrate DAPD can be applied to detect outliers in HTPP datasets systematically.

### DAPD image segmentation out-performs other image segmentation methods

DAPD depends upon a novel image segmentation method that we developed to improve accuracy and applicability across new datasets compared with existing methods. The method depends on an algorithm that combines shape analysis and supervised machine learning. We benchmarked the accuracy of the DAPD image segmentation algorithm on public datasets (A1, A2, A3, A4) and our in-house generated dataset using dice, precision, recall, and Jaccard metrics (Table 1) [15, 16]. These datasets contain ground-truth RGB and binary images of Arabidopsis and tobacco plants. DAPD performed consistently well in all metrics across all 5 datasets. Notably, performance on the tobacco images (A3) comparable to performance on the Arabidopsis images, indicating DAPD is adaptable to plants with different structure.

**Table 1:** Accuracy results of the DAPD image segmentation algorithm on 4 public datasets and our dataset

Dataset	Accuracy (%)			
	Precision	Recall	Jaccard	Dice
A1	92.05 (3.65)	96.87 (4.81)	89.26 (4.43)	94.26 (2.59)
A2	90.90 (8.60)	97.45 (6.76)	88.83 (10.43)	93.71 (6.69)
A3	95.86 (5.14)	93.88 (14.48)	89.85 (13.85)	93.86 (10.97)
A4	94.89 (4.29)	98.37 (2.50)	92.56 (5.09)	96.06 (2.87)
Our dataset	96.05 (5.42)	98.57 (1.61)	93.29 (2.98)	96.58 (1.64)
Mean	93.95 (5.42)	97.03 (6.03)	90.76 (7.36)	94.90 (5.78)

The numerical values in each cell represent the mean and standard deviation (in parentheses). The last row indicates the overall performance of our algorithm

We compared the performance of the DAPD image segmentation algorithm with 3 others (Table 2); the Rosette Tracker algorithm [17], the probabilistic parametric active contours algorithm [18], and the Image-based plant phenotyping with incremental learning and active contours [15]. The comparison was conducted by running all 5 algorithms on the same datasets (A1, A2, A3, A4, and our dataset) and calculating the mean of each performance metric. The rosette tracker and active contours algorithms had high recall metrics (99.97% and 99.66%, respectively) but low precision (54.29 and 42.25%), indicating a high false-positive rate in their predicted segmentation results. In contrast, DAPD image segmentation strongly outperformed active contours and rosette tracker and made notable improvements over incremental learning. DAPD had the highest precision and recall values, demonstrating it returned the most accurate results (precision = 93.95%) as well as returning the most positive outcomes (recall = 97.03%). The improved performance of DAPD segmentation compared with other methods may be because DAPD utilizes a combination of multiple image processing algorithms, such as K-means, image histogram analysis and shape analysis [11–13]. DAPD selects and applies the most suitable processing algorithm using a decision tree algorithm dependent on image features.

**Table 2:** Accuracy comparison between active contours, rosette tracker, incremental learning, graph segmentation, and DAPD algorithm

Method/algorithm	Accuracy (%) (A1, A2, A3, A4 and our dataset)			
	Precision	Recall	Jaccard	Dice
Active contours	42.25 (26.05)	99.66 (26.18)	42.19 (25.66)	59.34 (26.20)
Rosette Tracker	54.29 (18.11)	99.97 (17.79)	54.29 (19.22)	70.37 (15.00)
Incremental learning	89.87 (13.68)	91.94 (2.96)	83.90 (14.44)	89.72 (12.36)
DAPD	93.95 (5.42)	97.03 (6.03)	90.76 (7.36)	94.90 (5.78)

Each cell shows the mean and standard deviation of the 4 metrics (precision, recall, Jaccard, and dice) across the 5 datasets (A1, A2, A3, A4, and our dataset)

## Discussion

Traditional growth analysis in HTPP systems relies on sowing-time as the temporal start point. This assumption could lead researchers to believe, for instance, that application of an experimental treatment elicits changes in growth at a given time point. However, these analyses may be confounded by differences in germination time or true leaf emergence between genotypes.

We propose the DAPD method to control for temporal differences in development within plant phenotyping datasets. This method uses plant developmental stages to normalize the timeline of phenotyping measurements. We demonstrate the utility of DAPD to normalize rosette area measurements, but it can be similarly applied to normalize any phenotypic measurements. Furthermore, the analysis we present is on Arabidopsis plants, but DAPD could be used to normalize HTPP data from any species, with 2 contingencies: First, that a defined and relevant developmental scale can be provided to normalize to, and; second, that an algorithm is available to measure the phenotypic feature of interest in high throughput.

DAPD normalization improved the detection of outliers. Typically, a small proportion of plants develop abnormally in large-scale experiments. There are many causes, such as seed quality, low-frequency pathogen infection, or unintended stress. The DAPD method enabled clear, systematic detection of anomalous individuals (outliers) within datasets, which is not possible by inspecting central tendency metrics of the non-normalized datasets. DAPD includes a new algorithm to extract the rosette area and count the number of leaves from top-view images. This method outperformed other image segmentation methods when accuracy metrics were assessed on 5 different ground-truth datasets.

Comparisons between traditional analysis, where sowing time is taken as the reference point, and DAPD normalization illustrated that DAPD decreased dispersion of measurements. The difference in data dispersion between the 2 approaches gradually increased from the start of experiments to the end. This occurred because dispersion increased more rapidly over the plant lifecycle in the traditional (non-normalized) analysis than the DAPD normalized data. In principle, DAPD normalization will enable more sensitive detection of significant differences in trait values between plant lines or ecotypes, due to this decreased variance in measurements. DAPD normalization might also identify differences in germination phenotypes that might otherwise go unnoticed. For example, a mutant with an apparently greater rosette area than wild type during an early growth stage might either have a higher growth rate or germinate earlier. Assuming development was otherwise unchanged, DAPD normalization would eliminate the former possibility, allowing researchers to focus subsequent experiments.

## Conclusions

DAPD enables the normalization of HTPP datasets for temporal differences in development. This reduces the dispersion of data, thereby improving the detection of outlier plants and, in principle, the sensitivity with which differences in traits are detected. In principle, it can be applied to HTPP data from any species/trait combination for which a relevant developmental scale can be defined. Our code is available for reuse at https://github.com/diloc/DAPD_Normalization.git.

## Methods

### Plant material and growth conditions

Seeds were sterilized in a desiccator using chlorine gas for 150 min then stratified for 3 days in 0.1% agarose in the dark at 4 ºC. Afterward, the seeds were sown in vermiculite, perlite, and soil mixture (1:1:3), with 20 pots per tray. After germination, only a single seedling was retained per pot. The seedlings were grown in a controlled environment room at 20 ºC with 50% humidity and were watered with 500 ml water every 4 days after sowing. Illumination was with an LED light source; it used seven light wavelengths including near-infrared (850 ηm), far-red (740 ηm), deep red (660 ηm), red (618–630 ηm), green (530 ηm), blue (450–460 ηm) and cold white, supplied by PSI Instruments. The average irradiance output on the chamber was set at 150 μmol m^-2^ s^-1^ in the photosynthetically active radiation spectrum.

In experiment 1, 355 individual plants of Arabidopsis wild-type (Col-0) were grown under 12 h light / 12 h dark cycle. In experiment 2, 140 individuals each of Arabidopsis ecotypes Cvi and Col-0 were grown under long-day conditions (14 h of light/10 h of dark).

### Imaging: overview

**Fig. 6:**
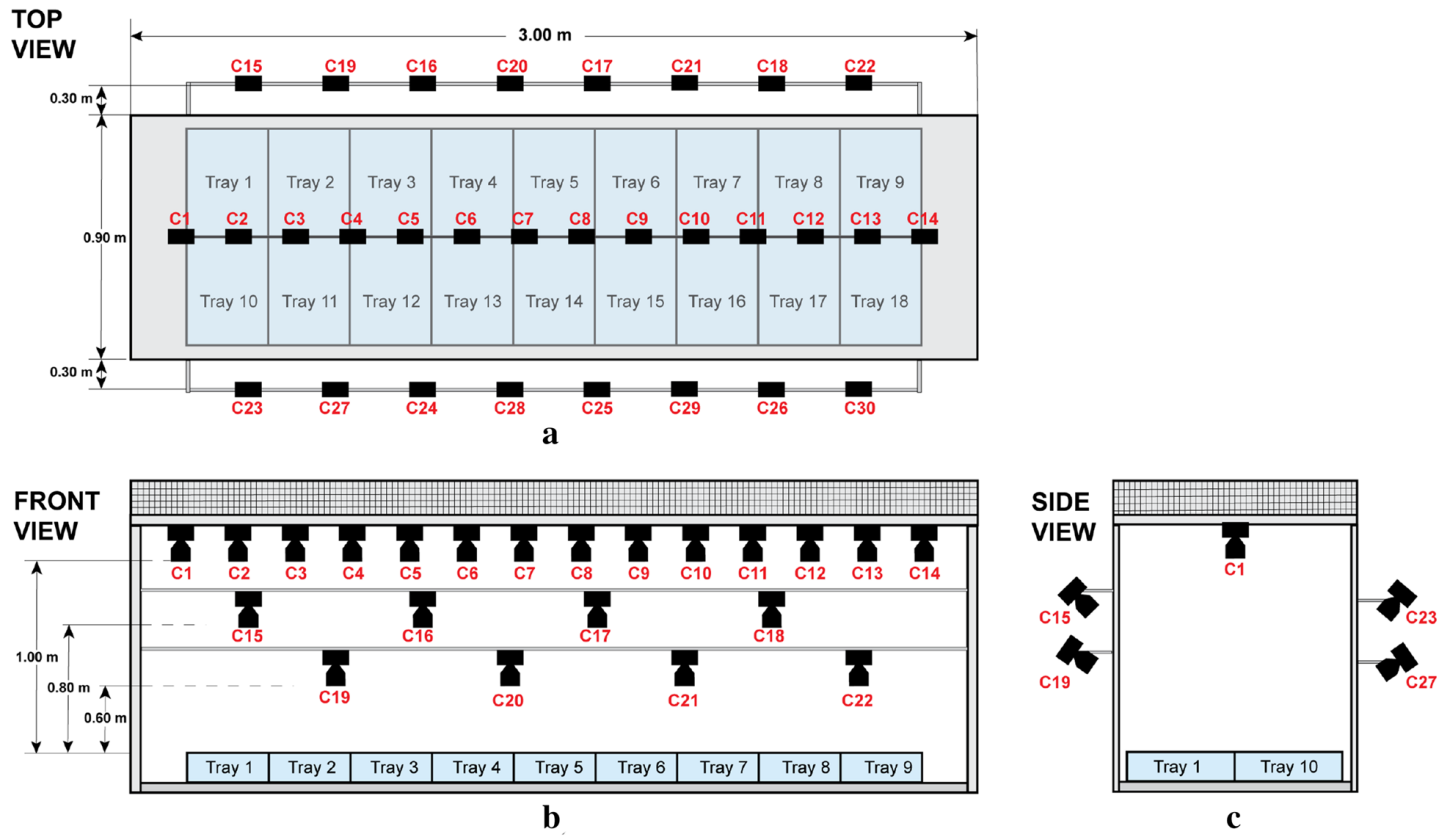
HTPP camera system. It consists of 30 cameras, the top view (**a**) shows camera distribution where 14 are placed along the central axis of the system and perpendicular to the trays. The side-cameras are distributed along the 2 longest sides of the system. The front view (**b**) shows that 14 cameras in the center are housed within the ceiling of the system, at 1 m from the height-adjustable tray surface during this experiment. Four side-cameras are located at 0.80 m and four side-cameras at 0.60 m. The side view (**c**) shows how the side-cameras are tilted to obtain an optimal focus point

**Fig. 7:**
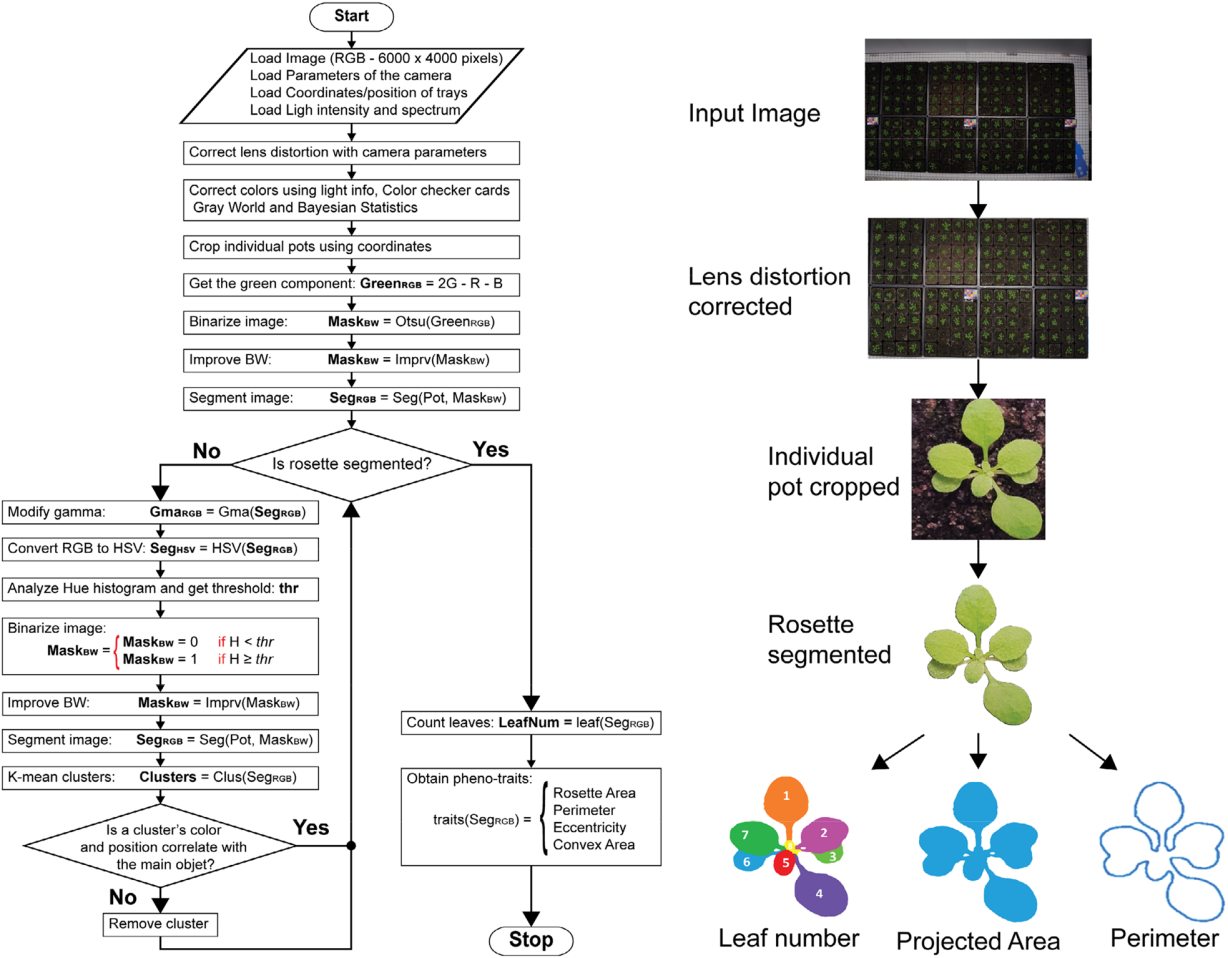
Flowchart documenting major steps in our image processing pipeline. First, RGB images, camera parameters, the spatial coordinates of the trays, and light information are loaded. Then, the image lens distortion is corrected using the camera parameters. The colors of the pot images are adjusted using the gray world method, and the light intensity and spectrum. Next, the individual pot images are cropped using the tray coordinates. Then, the rosette of the plant is segmented from the pot image by analyzing the hue under different gamma values. The final step of the image processing is to obtain the phenotyping traits from the segmented rosette image, such as rosette projected area, leaf number, and perimeter

The HTPP used a stereo vision system that had 30 fixed-cameras at different positions and angles in the setup (Fig. 6, Additional file 1. Figure S1). Images were acquired every 10–15 min during the daytime. The stereo camera system allowed depth information to be obtained by using multiple views of individual plants. The system was controlled by software that synchronized cameras and acquired images at the same time. After acquisition images were preprocessed to improve quality by reducing noise and correcting color and lens distortion. Subsequently, image segmentation algorithms extracted the rosette area and shoot phenotypic measurements such as the rosette area, growth rate, and leaf number were calculated. The image processing steps and details are described in Fig. 7.

### Image preprocessing

Image preprocessing algorithms improved the image quality by reducing noise and correcting the lens and color distortion. The noise could be unintentionally inserted into digital images at many stages, including acquisition, transmission, and storage [19]. We reduced the noise by applying image filters, including a median filter, which suppressed the salt-and-pepper noise and Gaussian filters, which reduces the high-frequency noise.

Color distortion deteriorated the image quality, and it was caused by the scene and illumination conditions, which was notable when using LED illumination [20]. We corrected the color distortion using the local illumination information, color checker cards, gray world method, and Bayesian statistics. First, the light color temperature at each pot region was estimated using the incident light spectrum and intensity distribution ( Additional file 1. Figure S2). The incident light spectrum and distribution were determined by taking 2,000-point measurements on a grid covering the apparatus imaging area using a SpectraPen LM500 (Photon Systems Instruments). Color-checker cards were included on 4 trays. The color checker cards were extracted from the image, and the color difference between their color in the image and their “true” color was calculated ( Additional file 1. Figure S3). We then calculated the contrast (α) and brightness (β) coefficients that best fit the color difference. These coefficients and the light intensity were used as a prior condition to calculate the likelihood of contrast and brightness in other parts of the image (pot regions) using Bayesian statistics. Then, local coefficients were used to correct the colors in these regions.

Lens distortion alters the scene geometry by curving and distorting the shape of objects in an image. It was corrected by applying the optimal rotation and translation camera parameters. These parameters were previously calculated by an OpenCV algorithm, which was based on chessboard pattern images and the pin-hole camera model [21]. The final step of preprocessing the image was to crop individual pots by using the spatial coordinates of trays and an adaptive window.

### Rosette segmentation

The projected rosette area of individual plants was extracted from preprocessed images by using a combination of multiple image segmentation algorithms. A decision tree algorithm was used to choose an image segmentation algorithm based on the pot image features. The image segmentation algorithms included K-means, image histogram analysis, and shape analysis [11–13]. The pot image features were obtained mainly from the HSV color space, especially, the hue (H) channel used to extract the visible light chromaticity features from it. The V channel was used to enhance the contrast of the picture by applying the Contrast Limited Adaptive Histogram Equalization (CLAHE) technique [22]. Subsequently, the green color features of the image were obtained by the independent contribution of the RGB (Green = 2G-R-B) and hue channel from the HSV color space. The green component image was binarized using Otsu’s method for global automatic thresholding [12]. The salt-and-pepper noise of the Otsu result was removed by applying a median filter, followed by using a Gaussian filter, which reduced high-frequency spatial noise. The binary image was further processed by an area-fill operation to remove small unwanted background regions or holes.

The resultant binary image area, hue, and green features were used to select a segmentation algorithm that could segment the plant shoot from the original RGB image. For instance, undesired background objects were identified and removed from it by grouping into coherent classes using K-means clustering [11]. Then, a histogram of the Hue channel was extracted and smoothed using the Savitzky-Golay filter [23]. A complementary spatial correlation test was used to determine the rosette objects. The test checked the correlation between image clusters. These clusters were formed by selecting all colors in distribution except by color values beyond the curve’s intersection point. Spatially correlated clusters belonged to the same object and were retained. Non-correlated clusters were removed. The whole procedure was repeated multiple times, and at each time, the gamma value was randomly modified.

This iterative approach occasionally over-segmented some rosette areas, but it was corrected by using Kriging [24]. Kriging is also known as Gaussian process regression, which produced predictions of unobserved values from observations at nearby locations [25]. Finally, the projected rosette area was obtained from the segmented rosette image. Unexpected power disruptions caused some image data not to be acquired during the time-series. Considering the complete time series as being images acquired every 15 min during the day, the data missing from experiment 1 was 14.83% of the total data, and in experiment 2 was 5.6%. To enable consistent downstream analyses, we applied data imputation techniques to estimate the missing data, including upsampling to a higher frequency, spline interpolation, and curve fitting.

### Individual leaf segmentation/counting (DAPD segmentation)

Our leaf segmentation approach combined 2 algorithms; edge contour extraction and marker-controlled watershed [13]. The edge contour extraction process was applied to the rosette binary image to obtain the rosette contour using topological analysis [14]. The center of mass of the rosette was calculated using the Hough transformation. This information was used to estimate leaf morphology features such as tip, base, center, and petiole. The watershed transformation was applied to the segmented rosette image, and centers of leaves were used as initial markers. This identified overlapping leaves and separated convex and smooth rosette features that touched. After applying the watershed transformation, the resulted number of markers represented the total number of leaves in the rosette.

### Depth maps

The depth information of plants was obtained by calculating the disparity between 2 or more images taken at the same time by cameras located at different distances and angles relative to the object plant. Stereo correspondence was calculated using a block matching method [26]. The depth maps were used to give more precise area information because they estimated the 3D surface area of the plant rosette rather than the 2D projections of plants on images. The 3D surface area measurements were less affected by normal plant nastic movement than the 2D projected area [27].

### Developmental normalization (DAPD normalization)

The normalization by development removed the time difference between plant measurements, which had similar developmental stages. These stages were identified by the leaf number based on the adjusted BBCH scale [3].

The leaf number of each plant was measured by applying the DAPD segmentation algorithm to the rosette images. The measured leaf number was a discrete function (1), which depended on the real leaf number, leaf occlusion effect, and modeling error denoted ME(t).

1 $${Leaf}_{meas}\left(t\right)={Leaf}_{number}\left(t\right)+{Leaf}_{occlusion}\left(t\right)+ME\left(t\right)$$

The variability of the measured leaf number function (1) was minimized by calculating the trend using curve fitting. This trend assembled an exponential function in the early developmental stages (2). The coefficient was the initial value of the function, and b was the growth rate.

2 $${Leaf}_{trend}\left(t\right)=a{e}^{bt}$$

The leaf number trend may vary from plant to plant within the same line/mutant population at a time point. Then, the average leaf number trend among all individuals was calculated to homogenize the trends (3).

3 $$\stackrel{-}{{Leaf}_{trend}\left(t\right)}=\frac{\sum_{i=1}^{N}{a}_{i}{e}^{{b}_{i}t}}{N}$$

The time-difference of plants with similar leaf number was removed by finding the best timeline in (1) that fitted (3). Multiple timelines were generated from the original timeline (t) by inserting a time delay (k). This time delay could be a positive or negative integer number that shifted the original timeline in days (t–k). The mean squared deviation (MSD) was calculated per each time delay (4). The time delay (s) that produced the lowest MSD was selected to shift the leaf number (5) and rosette area (6) time series. However, this time delay (s) must be adjusted because plants could have the same number of leaves, but the rosette area might be different due to the maturity and expansion of leaves. ∆t represented the time delay adjustment, as shown in Eq. (7).

4 $$MSD= \frac{1}{n}\sum_{i=1}^{n}{\left({ Leaf}_{meas}\left(t-k\right)- \stackrel{-}{{Leaf}_{trend}\left(t\right)}\right)}^{2}$$

5 $${Leaf}_{meas\_shift}\left(t\right)={ Leaf}_{meas}\left(t-s\right)$$

6 $${Area}_{meas\_shift}\left(t\right)={ Area}_{meas}\left(t-s\right)$$

7 $${Area}_{shift}\left(t\right)={ Area}_{meas}\left(t-s+\Delta t\right)$$

## Supplementary information

**Additional file 1: Figure S1.** Our HTPP system. **Figure S2. ** Distribution of the light intensity on the HTPP system. **Figure S3.** Color checker card comparison.

## Data Availability

Our code is available for reuse at https://github.com/diloc/DAPD_Normalization.git. Correspondence should be addressed to m.lewsey@latrobe.edu.au.

## Abbreviations

BASF: German for Baden Aniline and Soda Factory
BBCH: BASF, Bayer, Ciba-Geigy
CLAHE: Contrast limited adaptive histogram equalization
DAPD: Digital adjustment of plant development
DAS: Day-after-sowing
HSV: Hue, saturation, value
HTPP: High throughput plant phenotyping
LED: Light-emitting diode
RGB: Red, green, blue
