# Supplementary material for: Developmental normalization of phenomics data generated by high throughput plant phenotyping systems
Source: Plant Methods. 2020 Aug 12;16:111. doi: 10.1186/s13007-020-00653-x (PMC7424680; doi:10.1186/s13007-020-00653-x)
Supplement: Supplementary file 1 — Additional file 1: Figure S1. Our HTPP system. Figure S2. Distribution of the light intensity on the HTPP system. Figure S3. Color checker card comparison. [file 13007_2020_653_MOESM1_ESM.docx]

**Additional figures**

Figure S 1. Our HTPP system. The system can accommodate 18 trays of 20 pots (360 pots). The shelf can move up and down to adjust the distance from the light source. The plants are illuminated using LED lights that operate at different wavelengths. The system uses a stereo camera system (30 cameras) to acquire images at different positions and angles for calculating the depth of objects.

Figure S 2. Distribution of the light intensity on the HTPP system. (a) shows 2000 measurement points of the light intensity, which were close to the location of plants. The x-axis represents the length of the system, the y-axis, the width, and the z-axis the light intensity at each point. (b) shows the three-dimensional surface intensity distribution of the light. It was calculated using interpolation between neighboring points.


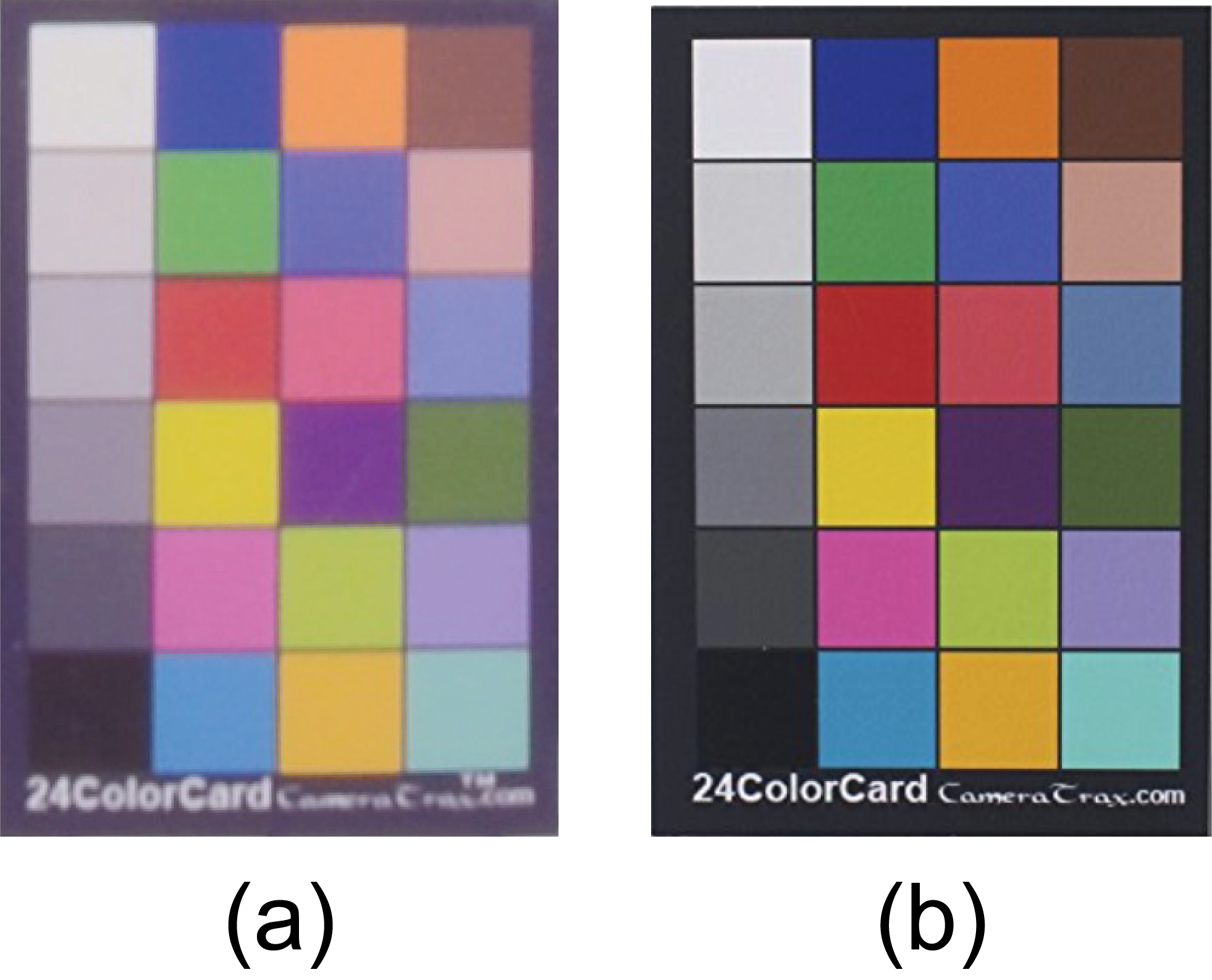


Figure S 3. Color checker card comparison. (a) represents the card extracted from the image and (b) the ideal color checker card.
